# Financing strategies for contract agricultural supply chain considering government subsidized interest

**DOI:** 10.1371/journal.pone.0341465

**Published:** 2026-02-24

**Authors:** Jialuo Wang, Changhong Li, Yifan Shi

**Affiliations:** School of Economics and Management, Shanxi University, Taiyuan, China; Czech University of Life Sciences Prague: Ceska Zemedelska Univerzita v Praze, CZECHIA

## Abstract

This paper examines the financing strategy of capital-constrained farmers (traditional banking financing or e-commerce platform financing) and the government’s subsidy strategy (whether to subsidize) in the contract agricultural supply chain. The optimal decisions, profits, and social welfare are compared and analyzed under different scenarios, and the hybrid financing model is further extended. The study found that when the probability of normal production is low, it is optimal for the farmer to choose bank financing. The farmer’s choice of bank financing or platform financing is more profitable than the hybrid financing strategy in all cases. The platform can provide a short-term interest-free financing strategy to ensure the production and marketing of agricultural products. This study provides guidance on how to choose the financing strategy for the capital-constrained farmer and how the government implements subsidy policies.

## 1 Introduction

The issue of agricultural production is a worldwide problem. Data shows that by 2050, global agricultural production is estimated to need to increase by 56 % in order to achieve food security [1]. Especially in recent years, the gap between China’s grain production and sales has remained large, and it is estimated that the annual grain deficit is growing by 1%. To offset this food gap, the future must rely on land and machinery to improve production efficiency [2]. For example, farmers in Changfeng County have significantly enhanced cost reduction and efficiency improvement in strawberry production through precise fertilization and medicine application using intelligent identification systems [3]. However, financial constraints remain a significant challenge to farmers’ production and must be addressed to maintain food security [4].

In the traditional financing mode, farmers maintain agricultural production through bank financing. According to a recent survey, banks remain the most common source of external financing [5]. However, the high cost of financial services in rural areas, the lack of collateral and low credit ratings of farmers have led to some farmers facing difficult and expensive financing [6].

With the development of online platforms such as Amazon and JD.com, farmers are able to bypass traditional distribution channels and sell fresh, high-quality produce directly customers in the end market via the platform [7–11]. This has not only alleviated the imbalance between supply and demand in the agricultural supply chain, but also improved, to a certain extent, the financing challenges that hindered agricultural development.

Different from banks that are unable to judge the credit risk and repayment ability of farmers, the platform is willing to provide financing services for farmers. It is because the platform can not only sign orders with farmers and set wholesale prices to maximize its own interests [12], but also access to the accurate sales data of farmers, which provides it with a greater degree of initiative [9]. For example, JD Finance provides financing for the production materials needed in the agricultural sector [13]. However, given the complex cooperative and competitive relationships in e-commerce agricultural supply chains, it is unknown how capital-constrained farmers choose between platform financing and bank financing.

In addition, in order to incentivize banks and platforms to loan money to farmers, the government implements an interest subsidy policy, which not only greatly reduces the threshold of financing for farmers, but also shares the loaning risks of financial institutions [14]. For example, Henan Province issued 133 million yuan of inclusive finance development funds in 2023 to support and guide the development of the agricultural economy [15]. How do government subsidies influence capital-constrained farmers to choose between platform financing and bank financing? And how does it affect the willingness of banks and platforms to load? How does it affect social welfare? These are all unknown. Thus, government subsidies add an interesting element to the study of farmers’ financing choices in agricultural supply chains.

Although existing studies have extensively explored issues such as agricultural supply chains, supply chain finance, and government agricultural subsidies, there is still a gap in integrated research considering the combined role of e-commerce platforms as both a sales channel and a financing support provider under the influence of government subsidy interest rate. Based on this, the paper innovatively integrates the core feature of "the e-commerce platform having dual functions of sales and financing" in the contract agriculture supply chain, and takes into account the impact of government subsidy interest rates. Considering the uncertainty in agricultural production, this paper focuses on the interaction mechanism between the financing choices of capital-constrained farmers, platform interest rates, and government subsidies, aiming to provide theoretical basis for decision-making by all parties. The following research questions are addressed in this study:

- Should the farmer chooses bank financing or platform financing? How do key parameters such as the probability of normal production and bank interest rate affect the financing choices of the farmer?
- What are the optimal interest rates for bank financing and platform financing under different scenarios?
- How do government subsidies and their intensity affect the farmer’s financing choices?

To answer these questions, this paper develops a game model in which the capital-constrained farmer needs to obtain initial capital through financing from the bank or the platform and sell their agricultural products to consumers via the e-commerce platform. Based on government subsidy and the farmer’s financing options, this paper study four different supply chain financing models: *NB*, *NP*, *IB* and *IP*. For each model, we determine the optimal wholesale price of the platform and the optimal planting technology level of the farmer. Then we further explore the optimal interesting rates and analyze the impacts of government subsidy policy on social welfare. On this basis, we extend the hybrid financing mode for the farmer to confirm the robustness of the conclusions.

The main insights and findings of this paper are as follows:

- The option of financing mode for farmers mainly depends on the interest rate and the probability of normal production. If the probability of normal production is high, farmers will choose based on the interest rate. Otherwise, farmers will choose the bank financing mode.
- Banks set the interest rate that takes into account the cost of farmers’ planting techniques, while platforms provide short-term, interest-free loans in order to stabilize production and sales in the agricultural supply chain.
- Under the government subsidy policy, it is optimal for farmers to choose platform financing when the probability of natural disasters and the bank interest rate bank interest rates are high. In the bank financing mode, there exists an optimal level of subsidy to maximize the overall benefits. In the platform financing mode, the government subsidy policy is consistently better than the no-subsidy policy.
- The adoption of bank financing or platform financing by farmers is always better than the hybrid financing mode, which further validates the robustness of the conclusions in the paper.

The structure of this paper is organized as follows: Sect 2 presents a comprehensive literature review. Sect 3 describes the problem and builds the model. Sect 4 deduces the equilibrium results and makes a comparative analysis of financing modes. Sect 5 analyzes the influence of government subsidies. Sect 6 extends the basic model. Finally, Sect 7 summarizes the conclusions and provides management insights. Proofs for all lemmas and propositions can be found in the S1 Appendix.

## 2 Literature review

The literature review in this section focuses on agricultural supply chains, supply chain finance, and government policy in agriculture. Each of them will be reviewed below, and the innovations of this paper will be emphasized.

### 2.1 Agricultural supply chains

Currently, research in the field of agricultural supply chains has extensively covered aspects such as cooperation and coordination of supply chains, operational optimization, risk control, and financing.

Specifically, in the area of cooperation and coordination, Israel et al. [16] explored the impact of agricultural supply chain capabilities, subsidy programs, and dynamic pricing on the economic feasibility of small farmers. Some scholars studied how to achieve coordination among supply chain members through aspects such as pricing, inventory, and revenue sharing [17], optimal production [18], and option contracts [19]. Based on this, in the aspect of operational optimization, Luo et al. [20] proposed a dynamic pricing based on traceability goodwill to optimize the operational efficiency and profitability of the supply chain. Additionally, some scholars studied the optimization effects of blockchain applications [21] and reverse logistics [22]. In the aspect of risk control, Liu et al. [23] explored how cross-border e-commerce enterprises can promote the sustainable development of the supply chain by optimizing supply disruption risks and quality control mechanisms. Balezentis et al. [24] proposed an agricultural supply chain feasibility analysis framework, providing indicators and paths for agricultural food supply chains to cope with disruptions such as those caused by the pandemic. In the aspect of financing, Li et al. [25] explored the conditions and roles of capital-constrained manufacturers, banks, and insurance companies in the application of agricultural insurance in the agricultural supply chain under different power structures. Qin and Cao [26] studied the impact of the optimal decisions of goal-oriented farmers on the performance of agricultural supply chains composed of capital-constrained small farmers and intermediary platforms.

However, although the aforementioned studies have promoted the development of agricultural supply chain theory in various aspects, few studies have examined the sales of e-commerce platforms, financing constraints, and government subsidies within the same framework. Based on the background that farmers can obtain financing from e-commerce platforms, this paper studies the financing strategy choices of capital-constrained farmers, the formulation of the optimal interest rate by the financing platforms, and the subsidy strategies of the government.

### 2.2 Supply chain finance

Supply chain finance serves as a key tool for optimizing agricultural resource allocation. The research subjects include banks, insurance companies, platforms, as well as capital-constrained farmers, merchants, or manufacturers, covering dimensions such as financing options, green emission reduction, and sustainability.

For instance, Li et al. [25] explored the conditions and roles of capital-constrained manufacturers, banks, and insurance companies in the agricultural supply chain under different power structures. Based on this, Shen et al. [27] found that different financing options of capital-constrained manufacturers would affect the competitive situation. Specifically, trade credit financing has an advantage in retail competition, and credit combination and dual trade credit financing can achieve a win-win situation. Lin et al. [28] considered the e-commerce platform as a financing provider and constructed a supply chain consisting of risk-averse farmers, an agricultural enterprise, and an e-commerce platform. The enterprise first decides how many farmers will provide guarantees, and then the platform sets loan interest rate for farmers with and without guarantees. In terms of financing options, Wang et al. [29] studied the preferences of online retailers similar to new shippers for bank financing and platform financing. The study found that active e-commerce platform financing can achieve coordination of supply chain financing, thereby generating a larger number of orders and participant profits than through bank financing. Moreover, even if online retailers have sufficient working capital, they are more inclined to use coordinated active e-commerce platform financing rather than reject any external financing. In the field of green finance, Wu et al. [30] found that manufacturers’ investment in emission reduction can achieve a win-win situation for supply chain output and emission reduction, and trade credit financing can further enhance the effect of the contract on the increase of output and emission reduction levels in the supply chain. Tseng et al. [31] constructed a sustainable supply chain financing model, adopted the fuzzy ideal solution similarity preference ranking method, and found that economic factors have a significant impact on other aspects, while distribution management policies are an effective means to enhance practical effectiveness.

Although the aforementioned studies have made significant progress and most of them have covered various financing entities and methods, they have failed to consider the role of government subsidies and have not investigated the differentiated impact of the financing choices of financially constrained farmers on the upstream and downstream decisions of the supply chain (such as platform pricing and farmers’ production inputs).

### 2.3 Government subsidies

Research has found that government agricultural subsidies can promote agricultural production and maintain the stable operation of rural economies [32], and are an effective policy that has attracted much attention from scholars. Currently, the subsidy policies come in various forms, such as cost subsidies, risk subsidies, price subsidies, environmental subsidies, and premium subsidies.

Specifically, Alizamir [33] studied two subsidy programs provided by the US government to farmers: price loss compensation (paying subsidies to farmers when the market price is lower than the reference price) and agricultural risk compensation (activating when farmers’ income is below a certain threshold). Fu et al. [34] studied the interaction between government fixed subsidy policies, agricultural risk subsidy policies, and farmers’ sustainable investment. Considering the uncertainty of agricultural output, Ray et al. [35] studied when and what intervention mechanisms the governments of developing countries should adopt: production material cost subsidies and indirect support, especially in the presence of farmer heterogeneity. Based on this, Chen et al. [36] considered production efficiency and studied the government’s input-oriented and output-oriented subsidy policies. In terms of subsidy recipients, Wu and Zhu [37] considered the best subsidy model for the government in the context of agricultural e-commerce sales: e-commerce or planting area. Considering agricultural environmental issues, Zhang et al. [38] studied the effects of three subsidy schemes in the agricultural supply chain: separate yield subsidies, environmental innovation subsidies, and mixed subsidies, and found that mixed subsidies can reduce pollution emissions, increase yield, improve enterprise profits, and enhance consumer surplus, which is a truly effective and feasible solution. In terms of agricultural insurance, Tsiboe and Turner [39] found that the demand for crop insurance is strongly influenced by premium subsidies.

Although the aforementioned studies offer valuable insights into the mechanisms and effects of subsidy policies, most treat such policies as exogenous variables to the supply chain finance system. They fail to integrate them within a comprehensive “financing-production-sales” supply chain framework, particularly overlooking how government interest subsidies influence decision-making by farmers and platforms.

### 2.4 The position of this study

Although the existing literature has studied issues related to agricultural supply chain, supply chain finance and government agricultural policy, there is relatively little research on integrating sales on e-commerce platforms and financing to support agriculture. Especially, studies considering the impact of different financing strategies on the decision-making of all parties in the contract agricultural supply chain under the influence of government subsidy interest rate are still in a blank state. This study makes the following three contributions to the related literature.

Compared with previous studies, the differences of this paper lie in the following aspects: First, it considers the realistic scenario of contract agriculture and e-commerce. Secondly, it studies the financing model choice of capital-constrained farmers. In addition to traditional banks, e-commerce platforms play an important role in assisting farmers with loans in the current agricultural financing mode. Finally, for the first time, the impact of the government’s interest subsidy policy on contract farming financing is considered. Table 1 summarizes the contributions of related studies and shows the differences between this study and previous work.

**Table 1:** Comparison of the related works.

Literature	Random yield	Planting technique	Financing choice	Financing policy	Key contributions
Qin et al. (2024) [26]	√				Interaction between two types of farmers and intermediary platforms: risk-neutral and goal-oriented.
Wang et al. (2023) [29]	√		√		Farmers choose sales and financing models through e-commerce platforms with corporate social responsibility.
Lin et al. (2024) [28]	√			√	Financing interest rate setting of farmers under financing guarantee policy.
Shen et al. (2020) [27]	√		√		Do cash-constrained manufacturers choose bank financing or retailer financing?
Lu et al. (2024) [40]	√	√	√		Digital technology enables the choice of financing mode in agricultural production.
Tsiboe et al. (2023) [41]				√	This paper empirically studies the agricultural insurance subsidy policy through the data of American agriculture.
Li et al. (2023) [25]	√				Interaction between agricultural insurance and power structure of agricultural supply chain.
Hu et al. (2024) [42]		√			The application of blockchain technology in agricultural sales.
This study	√	√	√	√	This paper researches the impact of government subsidies on farmers’ financing choices and planting techniques under production uncertainty.

## 3 Model description

**Fig 1:**
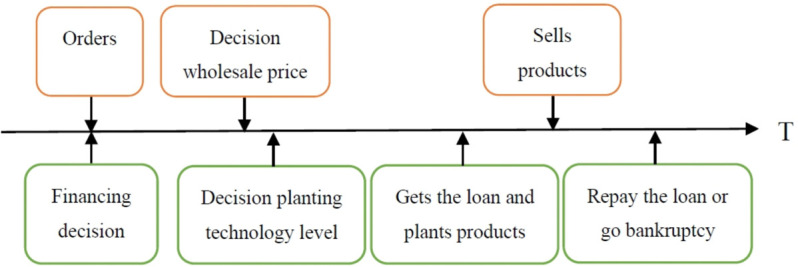
The sequence of events in operating and financing.

We develop a contract agricultural supply chain that consists of a capital-constrained farmer (labeled as *F*), a platform (labeled as *P*), and consumers. The platform signs orders with the farmer at a wholesale price per unit of *w*, and commits to purchase the entire output [43,44]. Based on this information, the farmer decides on the level of planting technology *e*_*F*_, such as scientific fertilization, modern irrigation, pest control, drone monitoring of agricultural products, etc. In addition, the farmer needs loans from the bank (labeled as *B*) or the platform to support their cultivation. Once the agricultural products are produced, they are sold by the platforms to downstream consumers at a retail price *p*. The farmer then repays the loan or declare bankruptcy, where *p* > *w*. The sequence of events in operating and financing is shown in Fig 1.

To ensure the rationality of the research, the model established in this study is based on the following assumptions. For more detailed mathematical information, please refer to the S1 Appendix.

**Assumption 1 (Yield randomness):** During the production of agricultural products, which is affected by uncontrollable factors such as floods and earthquakes, this paper assumes that the production of agricultural products per unit of land is a random variable and obeys the binomial distribution [45]. This assumption is not only commonly applied in the supply chain finance and agricultural supply chain fields, but also realistic in agricultural production activities. For example, in 2023, 600 acres of wheat in Zhumadian were damaged by rainy weather, resulting in granules [46], and corn granules in Northwest China were caused by drought and other natural conditions in 2021 [47].

**Assumption 2 (The yield of agricultural products):** This paper assumes that the yield of agricultural products is $Q=\left(1+\alpha e_{F}\right)xq$, where *α* represents the sensitivity of the farmer’s planting technology level to yield, and *q* represents the quantity of land owned by the farmer. Since the quantity of land owned by the farmer is basically constant from year to year, this paper considers it to be an exogenous parameter [40].

**Assumption 3 (The cost of financing):** We assume that the farmer has zero initial capital at the planting stage [48] and needs to obtain financing. There are two common ways of farmer financing. The traditional way of financing is from banks, with an interest rate of *r*_*B*_. The other is supply chain financing (platform financing) with an interest rate of *r*_*P*_. If the income of the farmer is not enough to pay the cost, at this time the farmer only needs to bear limited liability. We assume that the actual cost borne by the farmer is $\eta {e_{F}}^{2}\left(1+r_{B}\right)/2$ or $\eta {e_{F}}^{2}\left(1+r_{P}\right)/2$.

**Assumption 4 (The conditions that ensure production by farmers):** Due to the implementation of the national grain price stabilization policy [49], the retail price of agricultural products is relatively constant, which this paper considers exogenous. This assumption is commonly applied in the field of agricultural supply chain [50]. In the process of agricultural cultivation, if the cost of financing and the cost of planting technology are too expensive, farmers will not choose to put into production. In order to ensure that the agricultural supply chain can produce and sell normally, we assume that the retail price of agricultural products and the number of the farmer’s land satisfy a certain relationship. This paper investigates the optimal decision-making of farmers in the presence of the risk of bankruptcy.

**Fig 2:**
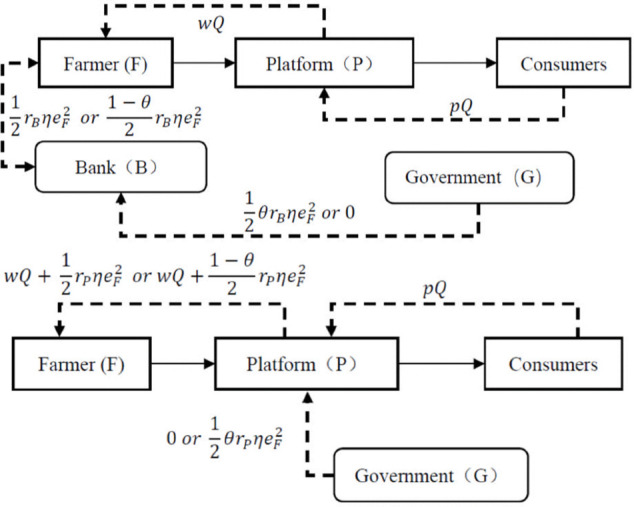
Contract farming supply chain financing models. A: Bank financing mode (*NB* or *IB*). B: Platform financing mode (*NP* or *IP*)

**Assumption 5 (Government interest subsidy):** In order to incentivize financial institutions to provide loans to the farmer actively, the government implements an interest rate subsidy policy to compensate the financial institutions for the loan interest at a proportion of *θ*. For example, Sichuan Province provides interest subsidies for agricultural working capital loans (such as grain production) at a rate of 150 basis points below the LPR [51]. The Shandong government reduces interest rate costs through subsidies, enabling the Dayang Town Cooperative to obtain loans to build 10 smart greenhouses, which has led to an annual income increase of 300,000 yuan [52]. The decision-making relationship among the farmer, the platform and the bank is as shown in Fig 2.

To summarize, the profits of the farmer under bank financing and platform financing without the subsidy policy are, respectively:

$$\pi_{F}^{NB}=E[wQ-\frac{1}{2}\eta {e_{F}}^{2}\left(1+r_{B}\right){]}^{+}$$ (1)

$$\pi_{F}^{NP}=E[wQ-\frac{1}{2}\eta {e_{F}}^{2}\left(1+r_{P}\right){]}^{+}$$ (2)

At this point, the platform’s profits under bank financing and platform financing without the subsidy policy are, respectively:

$$\pi_{P}^{NB}=E[\left(p-w\right)Q{]}^{+}$$ (3)

$$\pi_{P}^{NP}=E[\left(p-w\right)Q{]}^{+}+\frac{1}{2}\eta {e_{F}}^{2}\left(r_{P}\beta -\left(1-\beta \right)\right)$$ (4)

The profits of the farmer under bank financing and platform financing with the subsidy policy are respectively:

$$\pi_{F}^{IB}=E[wQ-\frac{1}{2}\eta {e_{F}}^{2}\left(1+\left(1-\theta \right)r_{B}\right){]}^{+}$$ (5)

$$\pi_{F}^{IP}=E[wQ-\frac{1}{2}\eta {e_{F}}^{2}\left(1+\left(1-\theta \right)r_{P}\right){]}^{+}$$ (6)

The parameters used in this paper are shown in Table 2.

**Table 2:** Summary of parameterization

Symbol	Definition
*q*	Quantity of land owned by the farmer
*α*	Sensitivity coefficients of planting techniques to yield
η	Cost sensitivity coefficients of planting technology
*p*	Retail prices of agricultural products
*w*	Wholesale prices of agricultural products
*β*	Probability of normal production, with 0≤β≤1
*θ*	Level of government interest rate subsidy, with 0≤θ≤1
*e*_*F*_	The level of planting technology
*r*_*B*_	Interest rates on bank financing, with $0\leq r_{B}\leq 1$
*r*_*P*_	Interest rates on platform financing, with $0\leq r_{P}\leq 1$
*SW*	Social welfare
$\pi_{i}^{k}$	Supply chain members *i*’s profit in strategy *k*.
**Subscript**	
i∈{F,P,B}	The farmer, the platform and the bank.
**Superscript**	
k∈NB,NP,IB,IB	The scenarios of strategy combination.

## 4 Model analysis

### 4.1 Financing model without government subsidy

This section discusses the optimal decision and profits under bank financing mode and platform financing mode respectively without government subsidy. It then compares and analyzes the results under different scenarios and further analyzes the optimal interest rates for financial institutions.

In the model of bank financing without government subsidy *NB*, the profit functions of the farmer, the platform and the bank are:

$\left\{ \begin{array}{c} \pi_{F}^{NB}=E[wQ-\frac{1}{2}\eta e_{F}^{2}\left(1+r_{B}\right){]}^{+}\\ \pi_{P}^{NB}=E[\left(p-w\right)Q{]}^{+}\\ \pi_{B}^{NB}=\frac{1}{2}\eta e_{F}^{2}\left(\beta r_{B}-\left(1-\beta \right)\right) \end{array}\right.$

By the method of backward induction, this paper obtains Lemma 1.

**Lemma 1.** *In the model NB, the equilibrium results are summarized as follows:*

- **The wholesale price:** $w^{NB*}=\frac{\left(pq\alpha^{2}-\eta -\eta r_{B}\right)}{2q\alpha^{2}}$
- **The level of planting technology:** $e_{F}^{NB*}=\frac{pq\alpha^{2}-\eta -\eta r_{B}}{2\alpha \eta (1+r_{B})}$
- **The profits:** $\pi_{F}^{NB*}=\frac{p^{2}q^{2}\alpha^{2}\beta }{8\eta (1+r_{B})}+\frac{\beta (2pq\alpha^{2}-3\eta -3\eta r_{B})}{8\alpha^{2}}$,$\pi_{P}^{NB*}=\frac{\beta {\left(pq\alpha^{2}+\eta +\eta r_{B}\right)}^{2}}{4\alpha^{2}\eta (1+r_{B})}$,$\pi_{B}^{NB*}=\frac{\left(\beta +\beta r_{B}-1\right){\left(pq\alpha^{2}-\eta -\eta r_{B}\right)}^{2}}{8\alpha^{2}\eta {\left(1+r_{B}\right)}^{2}}$.

According to Lemma 1, the farmer and the platform will produce and sell agricultural products properly only when the quantity of the farmer’s land satisfies $q>\frac{\eta (r_{B}+1)}{q\alpha^{2}}$. For the bank, the bank will earn profit only when the interest rate of the bank’s loan to the farmer satisfies $r_{B}>\frac{1-\beta }{\beta }$. Otherwise, the bank will refuse to provide loans to the farmer.

The derivation analysis shows that the price of wholesale agricultural products, the cultivation technology level, the profit of the farmer, and the profit of the platform increase with the increase of the retail price of agricultural products, the quantity of the farmer’s land and the sensitivity coefficient of the farmer’s cultivation technology. And they decrease with the increase of the coefficient of the cost of the cultivation technology and the bank interest rate.

In the model of platform financing without government subsidy *NP*, the profit functions of the farmer and the platform are:

$\left\{ \begin{array}{c} \pi_{F}^{NP}=E[wQ-\frac{1}{2}\eta e_{F}^{2}\left(1+r_{P}\right){]}^{+}\\ \pi_{P}^{NP}=E[\left(p-w\right)Q{]}^{+}+\frac{1}{2}\eta e_{F}^{2}\left(\beta r_{P}-\left(1-\beta \right)\right) \end{array}\right.$

**Lemma 2.** *In the model NP case, the equilibrium results are summarized as follows:*

- **The wholesale price:** $w^{NP*}=\frac{\beta \left(1+r_{P}\right)(pq\alpha^{2}-\eta -\eta r_{P})}{q\alpha^{2}\left(1+\beta +\beta r_{P}\right)}$
- **The level of planting technology:** $e_{F}^{NP*}=\frac{pq\alpha^{2}-\eta -\eta r_{P}}{\alpha \eta (3-\beta +\left(2-\beta \right)r_{P})}$
- **The profits:** $\pi_{F}^{NP*}=\frac{\beta^{2}(1+r_{P})(pq\alpha^{2}-\eta -\eta r_{P})(pq\alpha^{2}\beta +(2+\beta )\eta +\beta \eta r_{P})}{2\alpha^{2}\eta {\left(1+\beta +\beta r_{P}\right)}^{2}}$,$\pi_{P}^{NP*}=\frac{\eta (\beta +\beta r_{P}-1)}{2\alpha^{2}}+\frac{{\left(pq\alpha^{2}\beta +\eta \right)}^{2}}{2\alpha^{2}\eta (1+\beta +\beta r_{P})}$.

**Proposition 1.** *Comparison of the wholesale price, the level of planting technology, profit of the farmer and profit of the platform in the two financing modes without government subsidy.*

*(i) **The wholesale price:** if $pq\alpha^{2}\;-\;\eta \;+\;\eta r_{B}\;-\;2\eta r_{P}>0$, when $\beta_{1}<\beta <1$, w^NP^* > w^NB^*, when $0<\beta <\beta_{1}$, w^NP^* < w^NB^*; if $pq\alpha^{2}-\eta +\eta r_{B}-2\eta r_{P}<0$, w^NP^* < w^NB^*.*

*(ii) **The level of planting technology:** if $\eta_{1}<\eta <\overset{―}{\eta }$, $e_{F}^{NP*}<e_{F}^{NB*}$; if $\eta <\min\left\{\eta_{1},\overset{―}{\eta }\right\}$, when $\beta_{2}<\beta <1$, $e_{F}^{NP*}>e_{F}^{NB*}$, when $0<\beta <\beta_{2}$, $e_{F}^{NP*}<e_{F}^{NB*}$.*

*(iii) **The profit of the farmer:** if $0<r_{B}<r_{P}<1$, and $0<\eta <\eta_{2}$, when $0<\beta <\beta_{3}$, $\pi_{F}^{NP*}<\pi_{F}^{NB*}$, otherwise, $\pi_{F}^{NP*}>\pi_{F}^{NB*}$.*

*(iv) **The profit of the platform:** if $0<\eta <\eta_{3}$, when $0<\beta <\beta_{4}$, $\pi_{P}^{NP*}<\pi_{P}^{NB*}$, when $\beta_{4}<\beta <1$, $\pi_{P}^{NP*}>\pi_{P}^{NB*}$; if $\eta_{3}<\eta <\overset{―}{\eta }$, when $0<\beta <\beta_{4}$, $\pi_{P}^{NP*}>\pi_{P}^{NB*}$, when $\beta_{4}<\beta <1$, $\pi_{P}^{NP*}<\pi_{P}^{NB*}$.*

Proposition 1 indicates that the wholesale price under the bank financing mode is more expensive than under the platform financing mode if the probability of normal production is relatively small, or if the bank interest rate is relatively low. When the cost coefficient of planting technology is relatively high or the probability of normal production is relatively small, the planting technology level under the bank financing mode is higher than under the platform financing mode. The reason behind this is that at this point, the farmer needs to bear more cost or risk. When the farmer finances on the platform, the platform will consider its own interests and thus set a lower wholesale price, and the farmer’s incentive to increase production will be reduced, thus reducing the level of planting technology.

When the bank interest rate is lower than the platform interest rate, from the perspective of the farmer, if the cost sensitivity coefficients of planting technology and the probability of normal production are relatively low, the profit of the farmer under the bank financing mode is higher than under the platform financing mode. In addition, if the platform interest rate is lower than the bank interest rate, the platform financing mode is the optimal choice for the farmer. From the perspective of the platform, if the cost sensitivity coefficients of planting technology is relatively low and the probability of normal production is relatively high, the platform’s own profit is maximized under the platform financing mode. The reason behind this is that when the probability of normal production is relatively low, the farmer bears a higher risk of bankruptcy, and therefore, the farmer chooses the bank financing mode with lower interest rates, in which case the farmer’s expected return is maximized, and at this point the platform’s profit is maximized.

This conclusion is well verified in industrial practices and real cases. In Yanchi County, Ningxia, an arid region in Northwest China, the perennial rainfall is low and exposed to the risk of drought, so farmers have a low probability of normal production [53]. Against this background, farmers need stable financial support to respond to uncertainty and generally rely on loans from rural credit unions or rural commercial banks, such as the Agricultural Bank of China’s “Huinong Loan” [54]. Moreover, farmers used most of the credit funds for drought-resistant facilities (e.g., drip irrigation systems) and drought-resistant crop seeds, and only a small portion was used for expanding the scale of cultivation.

However, in Pingyang County, Zhejiang Province, the climatic conditions are relatively stable, and agricultural production is less disturbed by natural factors, so the probability of normal production is higher. Relying on the well-developed e-commerce infrastructure and perfect logistics network, the region has formed the characteristic development mode of “e-commerce + agriculture” [55]. In terms of financial support, backed by the Taobao ecosystem of the Netcommerce Bank plays an important role, Taobao transaction data and Alipay payment records become the basis for customer credit profiles, farmers can access more convenient sales of agricultural products and financing activities, and 80% of e-commerce sellers are using the Netcommerce Bank [56,57].

**Proposition 2.** *Optimal interest rate analysis of bank financing and platform financing.*

*(i) The optimal interest rate when the bank is profitable:*

*In order for the bank to earn a profit, it needs to fulfill the condition $r_{B}>\frac{1-\beta }{\beta }$. This means that the higher the probability of normal production, the lower the interest rate on bank agricultural loans.*

*If $0<\beta <(\frac{\sqrt{2}-\sqrt{10}}{4}{)}^{2}$, when $\eta <\frac{pq\alpha^{2}(1-\beta )}{2\beta }$, $r_{B}^{NB*}=\frac{\alpha \sqrt{pq\beta (pq\alpha^{2}\beta +8\eta )}-pq\alpha^{2}\beta -2\beta \eta }{2\beta \eta }$, when $\eta >\frac{pq\alpha^{2}(1-\beta )}{2\beta }$, $r_{B}^{NB*}=1$.*

*If $(\frac{\sqrt{2}-\sqrt{10}}{4}{)}^{2}<\beta <1$, when $\frac{pq\alpha^{2}(1-\beta )}{2\beta }<\eta <\frac{pq\alpha^{2}(1+\sqrt{2\beta })}{4}$, $r_{B}^{NB*}=1$, when $\eta >\frac{pq\alpha^{2}(1+\sqrt{2\beta })}{4}$ or $\eta <\frac{pq\alpha^{2}(1-\beta )}{2\beta }$, $r_{B}^{NB*}=\frac{\alpha \sqrt{pq\beta (pq\alpha^{2}\beta +8\eta )}-pq\alpha^{2}\beta -2\beta \eta }{2\beta \eta }$.*

*At this point, the bank’s maximum profit is:*


$$\pi_{B}^{NB*}=\{\begin{array}{cc} {}*20c\frac{{\left(3pq\alpha \beta -\sigma_{1}\right)}^{2}(\alpha \sigma_{1}-pq\alpha^{2}\beta -2\eta )}{16\alpha^{2}{\left(pq\alpha \beta -\sigma_{1}\right)}^{2}}, & r_{B}^{NB*}=\frac{\alpha \sigma_{1}-pq\alpha^{2}\beta -2\beta \eta }{2\beta \eta }\\ \frac{\left(2\beta -1)(pq\alpha^{2}-2\eta \right)}{32\alpha^{2}\eta } & ,r_{B}^{NB*}=1 \end{array}$$

*where $\sigma_{1}=\sqrt{pq\beta (pq\alpha^{2}\beta +8\eta )}$.*

*(ii) The optimal interest rate when the bank is losing money:*

*When $r_{B}<\frac{1-\beta }{\beta }$, the bank’s profit is negative, i.e., the bank is in a loss, then the bank’s optimal interest rate is $r_{B}^{NB*}=\frac{1-\beta }{\beta }$, when the bank can ensure that it minimizes its losses.*

*(iii) The platform’s optimal interest rate:*

*This paper concludes from the analysis of the platform profit function that when the farmer chooses the platform financing, the optimal interest rate is $r_{B}^{NB*}=0$, i.e., an interest-free loan.*

Proposition 2 indicates that the bank’s optimal interest rate has strong correlations with the probability of normal production and the cost sensitivity coefficients of planting technology. Among them, if the bank intends to earn a profit, the interest rate needs to satisfy $r_{B}>\frac{1-\beta }{\beta }$. While the platform in order to the supply chain can be normal production and sales, the determination of interest-free loan policy is its optimal choice.

In industrial practice, many financial institutions have adopted low-interest or even no-interest loan policies for farmers. For example, JD Finance, in cooperation with agricultural organizations, provides low-interest loans to loquat farmers in Renshou, Sichuan Province, relying on orders for the purchase of agricultural products, and provides liquidity loans to order farmers for their production needs [13]. For example, Netcommerce Bank provides interest-free loans to e-commerce merchants and farmers in 11 agricultural product counties in Zhejiang Province, including Pingyang County, to improve financial service support in the production, processing and sales of agricultural products and alleviate the financial pressure on farmers [58].

### 4.2 Financing model with government subsidies

In this section, the paper introduces the government’s implementation of the subsidy policy. Government intervention enables the farmer to maintain the production and market of agricultural products in a high-risk situation.

In the model of bank financing with government subsidies *IB*, the profit functions of the farmer, the platform and the bank are:

$\left\{ \begin{array}{c} \pi_{F}^{IB}=E[wQ-\frac{1}{2}\eta {e_{F}}^{2}\left(1+\left(1-\theta \right)r_{B}\right){]}^{+}\\ \pi_{P}^{IB}=E[\left(p-w\right)Q{]}^{+}\\ \pi_{B}^{IB}=\frac{1}{2}\eta {e_{F}}^{2}\left(\beta r_{B}-\left(1-\beta \right)\right) \end{array}\right.$

**Lemma 3.** *In the model IB case, the equilibrium results are summarized as follows:*

- **The wholesale price:** $w^{IB*}=\frac{pq\alpha^{2}-\eta -\eta (1-\theta )r_{B}}{2q\alpha^{2}}$
- **The level of planting technology:** $e_{F}^{IB*}=\frac{pq\alpha^{2}-\eta -\eta (1-\theta )r_{B}}{2\alpha \eta (1+(1-\theta )r_{B})}$
- **The profits:** $\pi_{F}^{IB*}=\frac{pq\beta }{4}+\frac{p^{2}q^{2}\alpha^{2}\beta }{8\eta (1+(1-\theta )r_{B})}-\frac{3\beta \eta (1+r_{B}-\theta r_{B}}{8\alpha^{2}}$,$\pi_{P}^{IB*}=\frac{\beta {\left(pq\alpha^{2}+\eta +(\eta -\eta \theta )r_{B}\right)}^{2}}{4\alpha^{2}\eta (1+(1-\theta )r_{B})}$,$\pi_{B}^{IB*}=\frac{(\beta +\beta r_{B}-1){\left(-pq\alpha^{2}+\eta +\eta r_{B}-\eta \theta r_{B}\right)}^{2}}{8\alpha^{2}\eta (1+(1-\theta )r_{B})}$.

Similar to Lemma 1, the bank is profitable when the interest rate of the bank satisfies $r_{B}>\frac{1-\beta }{\beta }$ under the government subsidy policy. According to the conclusion of Lemma 3, the wholesale price of agricultural products, the level of planting technology of the farmer, the profit of the farmer, the profit of the platform, and the profit of the bank increase with the increase in the level of the government subsidized interest rate.

In the model of platform financing with government subsidies *IP*, the profit functions of the farmer and the platform are:

$\left\{ \begin{array}{c} \pi_{F}^{IP}=E[wQ-\frac{1}{2}\eta {e_{F}}^{2}\left(1+\left(1-\theta \right)r_{P}\right){]}^{+}\\ \pi_{P}^{IP}=E[\left(p-w\right)Q{]}^{+}+\frac{1}{2}\eta {e_{F}}^{2}\left(\beta r_{P}-\left(1-\beta \right)\right) \end{array}\right.$

**Lemma 4.** *In the model IP case, the equilibrium results are summarized as follows:*

- **The wholesale price:** $w^{IP*}=\frac{\beta (1+(1-\theta )r_{P})(pq\alpha^{2}-\eta -\eta (1-\theta )r_{P})}{q\alpha^{2}(1+\beta +\beta (1-2\theta )r_{P})}$
- **The level of planting technology:** $e_{F}^{IP*}=\frac{\beta \left(pq\alpha^{2}-\eta -\eta (1-\theta )r_{P}\right)}{\alpha \eta (1+\beta +\beta (1-2\theta )r_{P})}$
- **The profits:** $\pi_{F}^{IP*}=\frac{\beta^{2}\eta {\left(1+r_{P}-\theta r_{P}\right)}^{2}}{2\alpha^{2}(1+\beta +\beta r_{P}-2\beta \theta r_{P})}+\frac{pq\beta (pq\alpha^{2}\beta +2\eta -2\beta \eta \theta r_{P})}{2\eta (1+\beta +\beta r_{P}-2\beta \theta r_{P})}$,$\pi_{P}^{IP*}=\frac{\beta^{2}(pq\alpha^{2}\beta +(2+\beta )\eta +\beta \eta (1-3\theta )r_{P})(1+(1-\theta )r_{P})(pq\alpha^{2}-\eta -\eta (1-\theta )r_{P})}{2\alpha^{2}\eta {\left(1+\beta +(\beta -2\beta \theta )r_{P}\right)}^{2}}$.

According to Lemma 4, when $0<\beta <\frac{1}{1+r_{P}}$, the wholesale price of agricultural products decreases with the increase in the level of government subsidy. When $\frac{1}{1+r_{P}}<\beta <1$, the wholesale price of agricultural products increases with the increase in the level of government subsidy. The level of planting technology, the farmer’s profit and the platform’s profit increase with the increase in government subsidy level.

**Proposition 3.** *Comparison of the equilibrium results of the wholesale price of agricultural products and the level of farming technology of farmers under the two financing modes in the case of government subsidy policy.*

*(i) If $0<\beta <\frac{1}{1+r_{P}}$ and $0<r_{B}<r_{B4}$, then w^IP^* < w^IB^*, otherwise, w^IP^* > w^IB^*.*

*(ii) If $\frac{1-\beta +\beta \left(1-2\theta \right)r_{P}}{2\beta \left(1-\theta \right)}<r_{B}<1$ and $\eta <\eta_{4}$, then $e_{F}^{IP*}>e_{F}^{IB*}$, otherwise, $e_{F}^{IP*}<e_{F}^{IB*}$.*

Proposition 3 shows that under the government subsidy policy, the wholesale price of agricultural products is significantly related to the probability of normal production and the loan interest rate. When the probability of normal production and the loan interest rate of bank are low, the wholesale price under the platform financing mode is higher than that under the bank financing mode, and in the rest of the cases, the wholesale price under the platform financing mode is higher. This is because when the probability of normal production is high, the production and sales of the agricultural supply chain are more stable, and farmers have a stronger ability to cope with risks, thus market competition is fierce. At this time, the platform occupies an advantageous position and raises wholesale prices in pursuit of profits. When the probability of normal production is low, the risk of supply chain disruption is large, and the platform lowers the wholesale price to stabilize the supply chain.

The level of farming technology of farmers is related to the bank interest rate and the cost coefficient. If the bank interest rate is relatively large and the cost coefficient is also relatively low, the level of planting technology under the platform financing model is higher than that under the bank financing model, and when the cost coefficient is relatively high, the level of planting technology under the bank financing model is higher. If the bank interest rate is relatively small, the level of planting technology under the bank financing model is higher. The reason for this is that when the cost coefficient is relatively low and the bank interest rate is very high, when the farmer chooses the platform financing mode, due to the relatively low cost coefficient, the farmer will improve the level of planting technology in order to increase production to obtain more profits.

**Fig 3:**
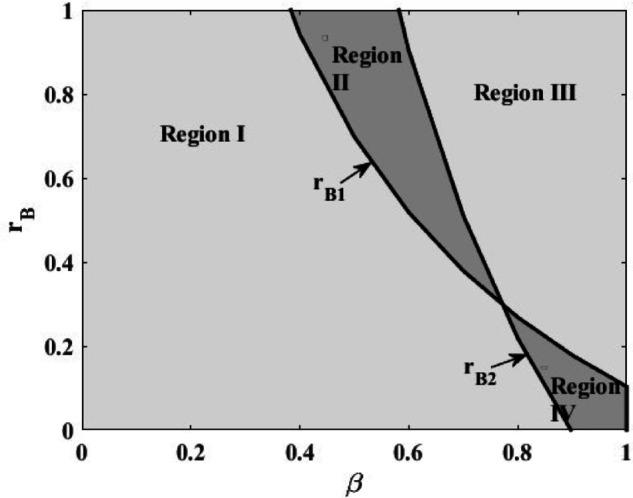
Financing model options under government subsidy policy.

Due to the complexity of the results, this paper analyzes the effects of bank interest rate *r*_*B*_ and farmer’s normal production probability *β* on farmer’s profit $\pi_{P}^{IB}$ and platform’s profit $\pi_{P}^{IP}$ through the method of numerical experiments (where $q=10,\alpha =1,p=5,\eta =10,r_{P}=0.3,\theta =0.5$), and obtains the optimal financing strategies in different scenarios, as shown in Fig 3.

In Region I, the bank financing mode is the optimal strategy for both farmers and the platform; in Region II, the bank financing mode is the optimal strategy for farmers, and the platform financing mode is the optimal strategy for the platform; in Region III, the platform financing mode is the optimal strategy for both the farmer and the platform; in Region IV, the bank financing mode is the optimal strategy for the platform, and the platform financing mode is the optimal strategy for farmers. Where *r*_*B*1_ stands for $\pi_{P}^{IB}=\pi_{P}^{IP}$, *r*_*B*2_ stands for $\pi_{F}^{IB}=\pi_{F}^{IP}$.

Fig 3 shows that the higher the probability of normal production and the bank loan interest rate, the platform financing mode is a better strategic choice relative to the farmer and the platform. The reason for this is that when the probability of normal production is higher, the risk of disruption of the agricultural supply chain is lower, so when the bank interest rate is higher, the farmer will choose the platform financing mode from the perspective of cost, and the platform can earn more profit in this case. Interestingly, when the bank interest rate is less than the platform interest rate, the farmer’s financing at the bank also has a positive effect on platform profits. The reason for this is that when the bank interest rate is lower, the farmer’s cost pressure will be greatly reduced and they will invest more in the level of planting technology, which finally increases the platform’s profit by increasing production. And through proposition 3 can find that at this time w^IP^* > w^IB^*, the farmer tend to choose platform financing mode.

### 4.3 Impact of subsidy level

In this subsection, we discuss the impact of government subsidy policy on contract farming supply chain financing. That means comparing the equilibrium results and profits under the two financing modes before and after the implementation of the government subsidy policy.

**Proposition 4.** *Comparison of wholesale prices of agricultural products, the level of planting technology and profits before and after the implementation of government subsidy policy.*

*(i) w^IB^* > w^NB^*,$e_{F}^{IB*}>e_{F}^{NB*}$, $\pi_{F}^{IB*}>\pi_{F}^{NB*}$, $\pi_{P}^{IB*}>\pi_{P}^{NB*}$.*

*(ii) If $\frac{pq\alpha^{2}\left(\beta +\beta r_{P}-1\right)+2\eta \left(1+r_{P}\right)}{\eta r_{P}\left(1+\beta +\beta r_{P}\right)}<\theta <1$, then w^IP^* < w^NP^*, otherwise w^IP^* > w^NP^*. $e_{F}^{IP*}>e_{F}^{NP*}$, $\pi_{F}^{IP*}>\pi_{F}^{NP*}$, $\pi_{P}^{IP*}>\pi_{P}^{NP*}$.*

According to Proposition 4, it can be concluded that both farmers and the platform under the government subsidy model are more profitable than the model without government subsidy policy, while the wholesale price of agricultural products and the level of planting technology will also increase. This indicates that after the government reduces the financing pressure on the farmer, the farmer will invest more in the level of planting technology, thus increasing the output of agricultural products, and finally improving the overall profit of the agricultural supply chain. This is similar to the findings of [26]. This finding has also been verified in practice. For example, in Yan’an, Shaanxi Province, bank fixed asset investment borrowing for the apple industry was subsidized at an annual interest rate of 3%. The support of subsidized loans allowed farmers to expand the scale of cultivation and improve production equipment, and the output of home orchards increased significantly and achieved better benefits. The policy not only reduces the financing costs of apple farmers and strengthens their confidence in development, but also leads to the prosperity of related industrial chains, such as processing and distribution, forming a superposition of comprehensive agricultural benefits [59].

**Fig 4:**
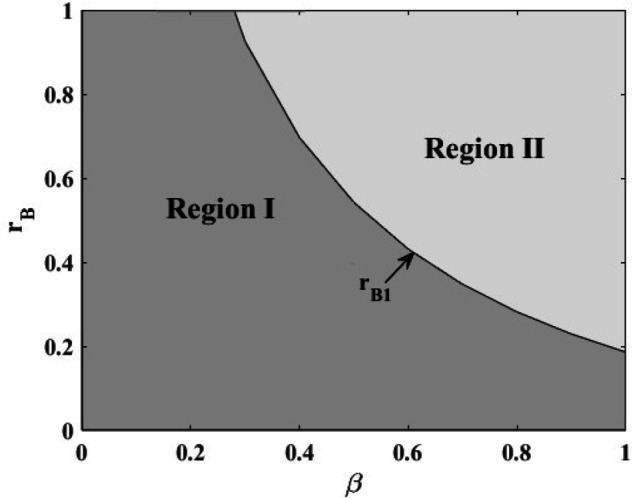
The impact of subsidy level.

This paper applies numerical experimental measures ($\alpha =1,q=10,p=5,\eta =10,r_{P}=0.3,\beta =0.9$) to discuss the impact of the level of subsidy on the effect of government subsidy policy under the two modes of bank financing and platform financing. Specifically, this paper measures the actual effect of the subsidy policy by comparing the level of farmers’ profits under without government subsidy mode and the relationship between farmers’ profits and government expenditures under government subsidy mode, as shown in Fig 4.

Through observation Fig 4, it can be concluded that under the bank financing mode, as the level of government subsidy increases the effect of government finance increases firstly and then decreases, so there exists an optimal level of government subsidy. Under the platform financing mode, the effect of government subsidy is always better than that in the case of no government subsidy policy. This shows that the effect of government subsidy policy under supply chain financing mode is more optimal, in general, no matter which financing mode is adopted by the farmer, there exists an optimal level of government subsidy, which is also verified by the fact that the government subsidy policy for the farmer is set to be around 0.5 in the real agricultural production process. Through observation, it can be concluded that under the bank financing mode, the effect of government subsidy first increases and then decreases as the level of subsidy increases. Under the platform financing mode, the government subsidized interest policy always brings gains to farmers’ profits. On the whole, no matter which financing mode is adopted by farmers, there exists an optimal level of government interest subsidy. This finding is also verified by the fact that in the real agricultural production process, the government subsidized interest level is set around 0.3-0.7. For example, Yiwu has introduced a grain production loan for grain growers who meet the conditions for interest rate subsidy, and the government subsidizes the loan at a rate of 3% and no more than 70% of the actual loan interest rate [60]. For example, Shanxi Province, engaged in food production, processing and other aspects of the main body eligible for interest subsidies, according to the actual loan interest rate of 40% to be subsidized [61].

In addition, although interest subsidies can reduce farmers’ financing costs, encourage agricultural production inputs, and guide financial and social capital to the agricultural sector, excessive subsidies have their risks and drawbacks. On the one hand, although subsidies have a positive effect on incentivizing financial institutions to serve farmers, an excessively high proportion of interest subsidies may create significant fiscal pressure and affect the long-term sustainability of policies. On the other hand, excessive subsidies may cause farmers or platforms to become policy-dependent, weakening their internal development momentum and ability to independently cope with market risk, thereby reducing overall supply chains efficiency. This suggests that policy making needs to focus on precision and flexibility, avoid "extensive" subsidies, and ensure that financial resources can guide supply chains towards healthy and self-sustaining development.

## 5 Social welfare

In this section, we analyze the influencing factors of social welfare under the four models. Then, we compare the social welfare to get the optimal level of government subsidy.

Social welfare includes the profits of all participants and consumer surplus, denoted by function SW=π+CS. According to Assumption 4, this paper takes into account the particularity of agricultural products, whose market prices are usually regulated by the national policy on stable grain prices. In the setting of this model, the retail price of agricultural products is regarded as an exogenously given parameter, rather than an endogenous variable determined by the platform. Therefore, consumer surplus remains unchanged. As a result, this paper only discusses the profits of enterprises and farmers in the study of social welfare. Under the without government subsidy mode and the government subsidy mode, respectively:

$\left\{ \begin{array}{c} SW^{N}=\pi_{F}^{N}+\pi_{P}^{N}\\ SW^{I}=\pi_{F}^{I}+\pi_{P}^{I}-G \end{array}\right.$

Where *G* is the government spending in the government subsidy mode, which is $\pi_{G}^{IP}=\frac{\eta e_{F}^{2}r_{P}\theta }{2}$,$\pi_{G}^{IB}=\frac{\eta e_{F}^{2}r_{B}\theta }{2}$ in the bank financing mode and platform financing mode, respectively.

**Lemma 5.** *The social welfare of the two financing modes under the without government subsidy mode and the government subsidy mode, respectively:*

$$SW^{NB*}=\frac{3p^{2}q^{2}\alpha^{2}\beta }{8\eta \left(1+r_{B}\right)}+\frac{\beta (6pq\alpha^{2}-\eta -\eta r_{B})}{8\alpha^{2}}$$ (7)

$$SW^{NP*}=\frac{pq\beta (pq\alpha^{2}\beta +2\eta )(1+2\beta +2\beta r_{P})}{2\eta {\left(1+\beta +\beta r_{P}\right)}^{2}}-\frac{\beta^{2}\eta {\left(1+r_{P}\right)}^{2}}{2\alpha^{2}{\left(1+\beta +\beta r_{P}\right)}^{2}}$$ (8)

$$SW^{IB*}=\frac{3pq\beta }{4}+\frac{3p^{2}q^{2}\alpha^{2}\beta }{8\eta \left(1+\left(1-\theta \right)r_{B}\right)}-\frac{\beta \eta \left(1+\left(1-\theta \right)r_{B}\right)}{8\alpha^{2}}-\frac{\theta r_{B}{\left(pq\alpha^{2}-\eta -\left(1-\theta \right)\eta r_{B}\right)}^{2}}{8\alpha^{2}\eta {\left(1+\left(1-\theta \right)r_{B}\right)}^{2}}$$ (9)

$$SW^{IP*}=\pi_{P}^{IP*}+\pi_{F}^{IP*}-\frac{\beta^{2}\theta r_{P}{\left(-pq\alpha^{2}+\eta +\eta r_{P}-\eta \theta r_{P}\right)}^{2}}{2\alpha^{2}\eta {\left(1+\beta +\beta r_{P}-2\beta \theta r_{P}\right)}^{2}}$$ (10)

**Fig 5:**
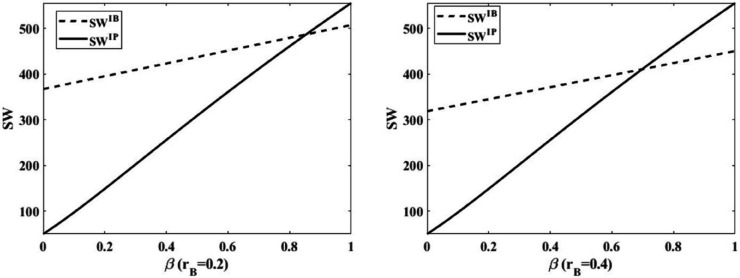
Comparison of social welfare of Model NB and Model NP.

**Fig 6:**
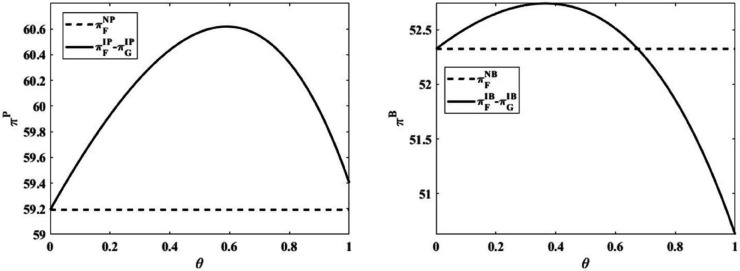
Comparison of social benefits of Model IB and Model IP.

This paper analyzes the optimal financing strategy from the perspective of social welfare maximization by means of numerical experiments (where $q=10,\alpha =1,p=5,\eta =10,r_{P}=0.3$), and obtains Figs 5 and 6.

As Fig 5 shows the optimal financing strategy under the without government subsidy mode. And we consider the impact of the probability of normal production *β* and the bank loan interest rate *r*_*B*_ on the optimal financing strategy. Region I represents SW^NB^* > SW^NP^*, Region II represents SW^NB^* < SW^NP^*, and *r*_*B*1_ represents $SW^{NB*}=SW^{NP*}$. The conclusion is obtained that it is optimal to select platform financing if both the probability of normal production and the bank loaning rate are high, and it is optimal to select bank financing in other cases.

As Fig 6 shows the impact of the probability of normal production *β* and the bank loan interest rate *r*_*B*_ on social welfare under the government subsidy mode. The higher the probability of normal production, the greater the social welfare under both financing modes. When the probability of normal production is higher, social welfare is higher under the platform financing mode than the bank financing mode. The social welfare is higher when the bank loan interest rate is lower than when the bank loan interest rate is higher. This is because the higher the bank interest rate, the higher the risk for the farmer, the farmer will reduce the input of planting technology, which leads to the reduction of production and finally leads to the reduction of social welfare.

## 6 Extensions

There is a third strategy that the farmer can adopt in addition to bank or platform financing, i.e., financing from both banks and platforms at the same time. In this section, we consider that the farmer raises funds from both banks and platforms, which is referred to as the Hybrid Model in the following. We assume that the farmer finances from the bank in proportion ρ and from the platform in proportion 1−ρ. We denote by *NH* when the government does not subsidize the financing interest and *IH* when the government subsidizes the financing interest. The detailed model specifications for farmers, platforms, and social welfare are provided in the S1 Appendix.

**Fig 7:**
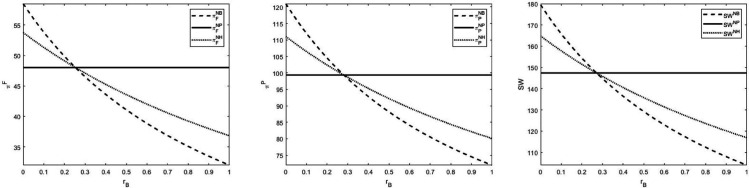
Comparison between Modes NB, NP, NH.

**Fig 8:**
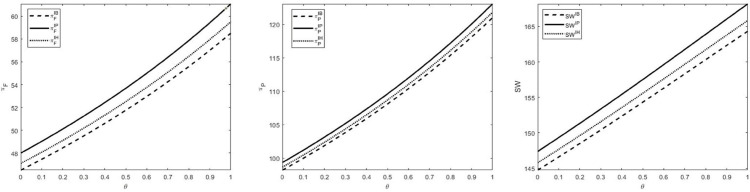
Comparison between Modes IB, IP, IH.

In this paper, we compare the profits of the farmer, the platform and the social welfare under three financing models by means of numerical experiments to explore whether farmers should consider hybrid mode, and obtains Fig 7 and Fig 8. The main consideration is whether the farmer should consider the hybrid model when financing. It can be found that regardless of whether the government provides loan subsidies on the farmer’s loans or not, the optimal financing mode option for the farmer is still the bank financing or the platform financing, and the hybrid financing mode will not increase the farmer’s profit, the platform’s profit and the social welfare. The conclusion that the mixed financing strategy is not as efficient as the pure strategy seems counterintuitive at first glance, especially since the mixed strategy often combines the advantages of different financing channels. However, in this case, the pure strategy is more advantageous. This is because when farmers adopt a mixed strategy, the platform bears part of the risk when it is high and loses part of the income when it is low, thereby reducing overall profitability. In addition, the mixed strategy also led to higher wholesale prices, thereby reducing farmers’ profits. In short, although the mixed strategy may seem to provide greater flexibility or risk management, in this particular case, it has the disadvantage of inefficiency. The pure strategy financing method is more profitable when we consider the limited liability of farmers and the platform’s pursuit of profit maximization.

## 7 Conclusions

Based on the output uncertainty of agricultural products, this paper develops a contract farming supply chain model consisting of the capital-constrained farmer and a platform, and considers the impact of government subsidy policy on the choice of financing strategy of the farmer. Firstly, the bank financing and platform financing models without government subsidy are established. Secondly, the bank financing and platform financing models with government subsidy are established. Finally, the model of the farmer’s hybrid financing from the bank and the platform is extended. The optimal financing model of the farmer and the impact of government subsidy policy on the contract farming supply chain are studied through comparative analysis, and the following conclusions are obtained:

(1) The farmer adopts bank financing strategy when they are more affected by the risk of natural disasters, while the platform’s profit under the two financing modes depends on the cost coefficient of planting technology and the probability of normal production. When the probability of normal production is relatively large and the cost coefficient of planting technology is relatively small, the platform’s profit in the case of platform financing is greater. Otherwise, the platform’s profit is greater in the case of bank financing. The bank financing or platform financing is still the optimal option for farmers relative to the hybrid financing, i.e., farmers will not increase their own profit or the overall profit of the platform and the supply chain by financing from the bank and the platform at the same time.

(2) When the bank’s interest rate is set to be greater than the ratio of the probability of abnormal production and the probability of normal production, the bank can be profitable and the optimal interest rate depends on the relationship between the probability of normal production and the cost of planting technology. And the platform’s optimal interest rate in the case of normal production and sales in the agricultural supply chain is zero, which verifies that the current agricultural supply chain is mostly characterized by the phenomenon of interest-free loans for supply chain financing, i.e., the platform retailer stabilizes the production and sales of the agricultural supply chain by providing financing services to the farmer who are short of funds.

(3) The magnitude of social welfare under the two financing modes of the contract farming supply chain depends on the relationship between the probability of normal production and the bank loaning rate. When the probability of normal production and the bank interest rate are both large, the social welfare under platform financing is greater than that under bank financing, otherwise, the social welfare under bank financing is greater. The government subsidy policy can enhance the profits of farmers and the platform under both bank financing and platform financing modes, but from the perspective of farmers’ profits and government expenditures, there exists an optimal level of interest rate subsidy for the government to optimize the implementation of the subsidy policy under both financing modes.

Based on the above research conclusions, the following management implications can be drawn: (1) For farmers, when production stability is relatively high, they should be courageous to take advantage of the channel, capital and resource advantages of e-commerce platforms, and ride on the entire industrial chain from production to sales to reduce the risks of facing the market independently and achieve stable income growth; (2) For e-commerce platforms, they should gradually shift their service focus from profit margins to the overall value enhancement of the supply chain, and build a comprehensive chain from agricultural product production to sales and financing, achieving sustainable business returns through stable supply and win-win cooperation; (3) Government subsidies can indeed promote the profits of all parties in the supply chain, but it is not the case that the higher the subsidy, the better. A reasonable level of interest subsidies should be set. At the same time, the government should further guide e-commerce platforms to assist farmers, promote inclusive rural finance, and help agricultural products in vast rural areas build efficient upward channels.

This study has certain limitations. First, the model presented here concentrates on short-term operational decisions, neglecting long-term evolution and development. Second, beyond government subsidies, numerous other significant factors can substantially influence supply chain coordination in the agricultural supply chain’s production and sales processes. Future research could expand in two key areas: First, analyze the multi-stage dynamic game between the government and the supply chain, examining the evolution of each participant’s strategies. Second, investigate the impact of agricultural insurance and digital technologies, such as blockchain and the Internet of Things, on financing risk management, cooperative coordination, and decision-making within agricultural supply chains.

## Supporting information

S1 Appendix Supplementary explanations for the model description, as well as the proof processes of all lemmas and propositions in the article.
(TEX)
